# Native Ambient Mass Spectrometry of an Intact Membrane Protein Assembly and Soluble Protein Assemblies Directly from Lens Tissue

**DOI:** 10.1002/anie.202201458

**Published:** 2022-06-21

**Authors:** Oliver J. Hale, Helen J. Cooper

**Affiliations:** ^1^ School of Biosciences University of Birmingham Edgbaston B15 2TT UK

**Keywords:** Ambient Mass Spectrometry, Aquaporin, Mass Spectrometry Imaging, Membrane Protein, Native Mass Spectrometry

## Abstract

Membrane proteins constitute around two‐thirds of therapeutic targets but present a significant challenge for structural analysis due to their low abundance and solubility. Existing methods for structural analysis rely on over‐expression and/or purification of the membrane protein, thus removing any links back to actual physiological environment. Here, we demonstrate mass spectrometry analysis of an intact oligomeric membrane protein directly from tissue. Aquaporin‐0 exists as a 113 kDa tetramer, with each subunit featuring six transmembrane helices. We report the characterisation of the intact assembly directly from a section of sheep eye lens without sample pre‐treatment. Protein identity was confirmed by mass measurement of the tetramer and subunits, together with top‐down mass spectrometry, and the spatial distribution was determined by mass spectrometry imaging. Our approach allows simultaneous analysis of soluble protein assemblies in the tissue.

## Graphical abstract

The integral membrane protein assembly Aquaporin‐0 was analysed by native ambient mass spectrometry of eye lens tissue. Membrane protein assemblies were solubilised by detergent micelles. Spatially‐resolved analysis for mapping protein assembly distributions, accurate intact mass determination, and the identification of assemblies approx. 100 kDa in molecular weight are demonstrated.

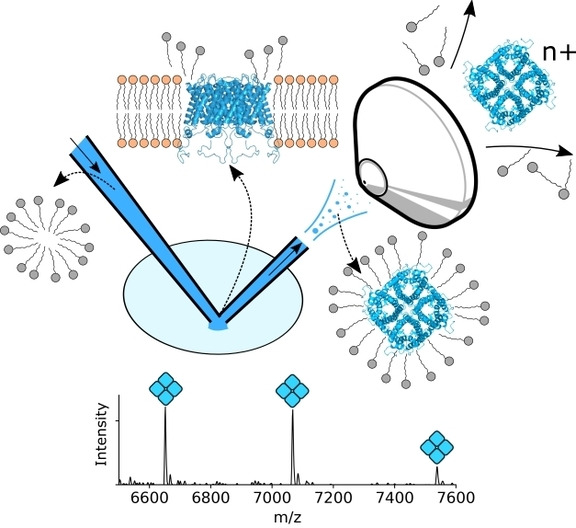

Native ambient mass spectrometry (MS) combines native MS,^[1]^ an established technique in structural biology, and ambient MS,^[2]^ in which biological substrates such as thin tissue sections are analysed directly with little or no sample preparation. The combined benefits of native ambient MS for the analysis of protein assemblies and protein‐ligand complexes include measurement of accurate mass and stoichiometry, identification of both protein and non‐covalently bound ligands, together with information on spatial distribution within the sample. Recent efforts using liquid extraction surface analysis (LESA)[^3^, ^4^] and nanospray‐desorption electrospray ionization (nano‐DESI), have advanced native ambient MS for the analysis of fresh frozen tissue, allowing the spatial analysis of protein assemblies up to 145 kDa in molecular weight.[^5^, ^6^] Endogenous protein assemblies and their constituents (including small molecule ligands) can be identified by top‐down dissection of assemblies in the gas phase, potentially allowing the discovery of new protein‐ligand interactions.[^6^, ^7^, ^8^] In addition, Sharon and co‐workers have demonstrated direct native mass spectrometry of crude cell lysates.^[9]^

To date, all protein assemblies analysed directly from tissue by native ambient MS have been soluble in aqueous solution. Yet membrane proteins, which are estimated to account for 30 % of the human proteome and 60 % of drug targets, are hydrophobic, which hinders their analysis by top‐down and bottom‐up proteomics approaches.[^10^, ^11^, ^12^] Methods incorporating strong detergents, which must be removed before MS analysis, or organic solvent systems that disrupt protein structure can improve membrane protein solubility but are incompatible with the aims of native ambient MS. Instead, a critical advance in the development of “standard” native MS was the ability to analyse intact integral membrane protein assemblies by their encapsulation within MS‐compatible detergent micelles, which simultaneously act as a membrane mimetic and solubilization aid.[^13^, ^14^, ^15^] Collisional activation within the mass spectrometer removes the micelles to reveal bare membrane protein ions in the gas phase. Using LESA, Mikhailov et al. analysed a purified membrane protein assembly after its deposition on a glass slide.^[16]^ Similarly, work by Ambrose *et al*. demonstrated the potential for analysing purified, intact membrane proteins directly from a glass surface using desorption electrospray ionisation (DESI).^[17]^ Recently, Chorev *et al*. forwent detergent assistance and were able to eject ionised membrane proteins directly from native membranes in the gas phase.^[18]^

Here, we demonstrate the analysis of an intact membrane protein assembly directly from tissue using nano‐DESI mass spectrometry. DESI and nano‐DESI are distinct ambient ionisation techniques, despite their similar nomenclature, as described by Laskin and co‐workers.^[19]^ We chose to work with eye lens tissue as it features an abundant homotetrameric integral membrane protein assembly, Aquaporin‐0 (Aqp0, also known as lens fibre major intrinsic protein, MIP), which has both structural and water transport roles *in vivo*.[^20^, ^21^] Each Aqp0 subunit (molecular weight 28.3 kDa, assembly mass 113.0 kDa) has six transmembrane helices and two intramembrane helices. Spatial analysis of denatured (i.e., monomeric) Aqp0 has been reported using matrix‐assisted laser desorption/ionization (MALDI) MS but required specific sample preparation and lacked quaternary structural information.[^22^, ^23^, ^24^] We report analysis of intact, tetrameric Aqp0; intact and subunit mass measurement, determination of complex stoichiometry, amino acid sequence information and spatial distribution throughout a lens section without sample preparation. As a non‐covalent integral membrane protein assembly and one of the highest mass assemblies analysed by nano‐DESI, its analysis provides conclusive demonstration of the methodology.

Figure 1a shows the procedure for intact membrane protein analysis directly from tissue: (1) The nano‐DESI solvent comprises an aqueous solution containing a concentration of MS‐compatible detergent greater than the critical micelle concentration (CMC), i.e., detergent micelles are present in the solution. Membrane proteins present in the tissue (2) are solubilized by encapsulation (3) in the detergent micelles. Micelle‐encapsulated membrane proteins are ionized and introduced to the mass spectrometer. Finally, energetic collisions with gas molecules in the mass spectrometer liberate membrane protein ions from the micelles (4). Bare membrane protein ions are then transmitted for *m*/*z* analysis.

**Figure 1:**
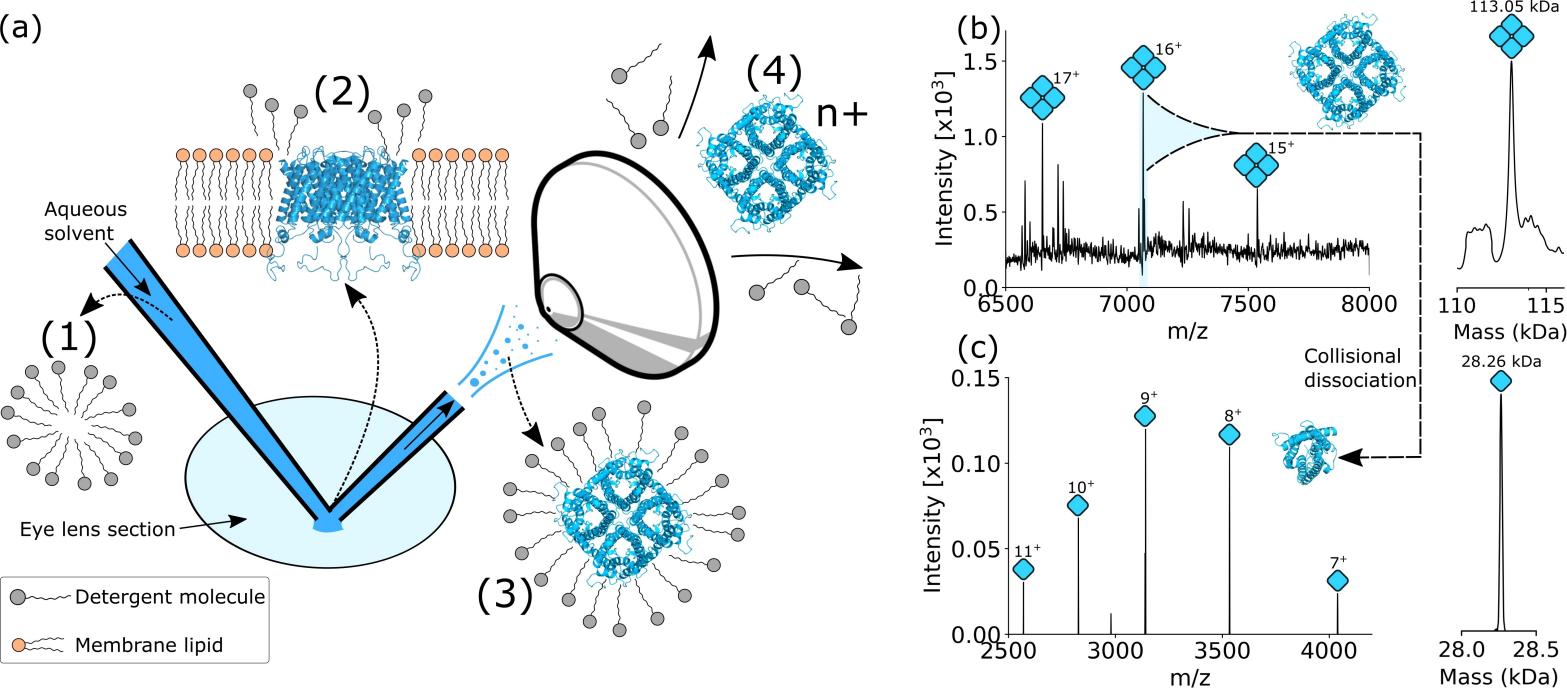
Nano‐DESI MS enables analysis of intact Aqp0 tetramers directly from eye lens tissue. a) Scheme depicting nano‐DESI sampling of eye lens for Aqp0 analysis; (1) solvent containing C_8_E_4_ detergent micelles is delivered to the tissue surface. (2) Aqp0 tetramers are extracted from cell lipid bilayers into detergent micelles in the liquid junction with the tissue. (3) Micelles containing intact Aqp0 tetramers are transferred into the gas phase, ionised, and transmitted into the mass spectrometer. (4) Energetic collisions with gas molecules in the mass spectrometer release charged, intact tetramer ions from the micelles. b) Full scan nano‐DESI mass spectrum showing Aqp0 tetramer signals corresponding to charge states 15+–17+ and the deconvoluted spectrum indicating an intact mass of 113.055 kDa. c) Isolation and collisional activation of the 16+ tetramer ions produced signals for Aqp0 monomers with mass 28.264 kDa, i.e., one‐quarter the mass of the intact assembly.

The approach outlined in Figure 1a was applied to the analysis of intact Aqp0 homotetramers directly from sheep eye lens tissue. The solvent system was 200 mM aqueous ammonium acetate containing 2× the CMC of the detergent octyl tetraethylene glycol ether (C_8_E_4_). The mass spectrum in Figure 1b reveals peaks corresponding to intact Aqp0 homotetramers in various charge states. The extended *m*/*z* range spectrum in Figure S1, Supporting Information, shows minimal dissociation of the tetrameric assembly on transmission through the mass spectrometer. Deconvolution of the spectrum in Figure 1b revealed the experimentally‐determined average mass to be 113 055 Da, in good agreement with the calculated mass of 113 051 Da. At 113 kDa, the Aqp0 assembly is one of the largest protein assemblies to have been analysed by native ambient MS. We also observed signals indicating phosphorylation of Aqp0 within intact tetramers (Figure S2, Supporting Information). Evidence of phosphorylation has previously been reported by bottom‐up proteomics and native MS of purified Aqp0.[^25^, ^26^] In the latter, Aqp0 subunits were determined to be singly‐phosphorylated by using surface‐induced dissociation (SID) to dissociate doubly‐phosphorylated tetrameric Aqp0 and leave post‐translational modifications intact. Signals indicating the presence of lipids associated with Aqp0 tetramers were not detected, likely due to the delipidating action of C_8_E_4_.[^27^, ^28^] Isolation of Aqp0 tetramer ions and subsequent collisional activation dissociated the tetramer into Aqp0 monomers (measured mass=28 264 Da, calculated mass=28 263 Da), see Figure 1c. Further confirmation of the intact mass of the tetramer and of the mass of the subunit were provided through proton transfer charge reduction (PTCR) mass spectrometry and high‐resolution MS (Figures S3 and S4 respectively, Supporting Information). Crucially, Aqp0 was not detected in the absence of detergent (Figure S5, Supporting Information).

Unambiguous identification of Aqp0 was achieved through fragmentation of the polypeptide backbone by multistage collisional activation (“pseudo‐MS^3^“). The resulting sequence fragment ions were searched against a sheep protein database, enabling identification of the protein (See Figure S6, Table S1, Supporting Information).

Our next step was to spatially map the distribution of Aqp0 tetramers in the eye lens section by use of nano‐DESI in mass spectrometry imaging (MSI) mode.^[29]^ Protein turnover in the eye lens is very low and so the distribution of proteins throughout the eye lens is indicative of their age; for example proteins in the human lens nucleus form around the time of birth and exist without turnover throughout life.^[30]^ Figure 2 shows the results from nano‐DESI MSI of a section of sheep eye lens tissue, with example mass spectra shown in Figure S7, Supporting Information. Replicate analyses are presented in Figures S8–S10, Supporting Information. An optical image of the lens section post‐analysis is shown in Figure 2a. The distribution of tetrameric Aqp0 (Figure 2b) shows that it was most abundant throughout lens cortex tissue, and of lower abundance within the lens nucleus. MSI of Aqp0 has been performed under denaturing conditions by MALDI MSI, (i.e., the unfolded monomers were imaged), which also indicated abundance within lens cortex tissue.^[22]^ Previous immunohistochemistry (IHC) analysis showed Aqp0 present in concentric rings in the lenses of mouse and rat;^[31]^ we detected a similar distribution in the lens in Figure 2b and the replicate analysis in Figure S8, Supporting Information, but not in another lens (see Figures S9 and S10, Supporting Information). Our lenses were from sheep reared for food, which are typically slaughtered at 6–7 months old in the UK but can range from 10 weeks to over 12 months.^[32]^ Thus, different Aqp0 distributions might be indicative of biological variance due to the age of the animal. Signals corresponding to the monophosphorylated tetramer (Figure S2, Supporting Information) showed greatest abundance between the two rings of unmodified Aqp0 tetramer, suggestive of age‐related post‐translational modification as the lens fibre cells age.

**Figure 2:**
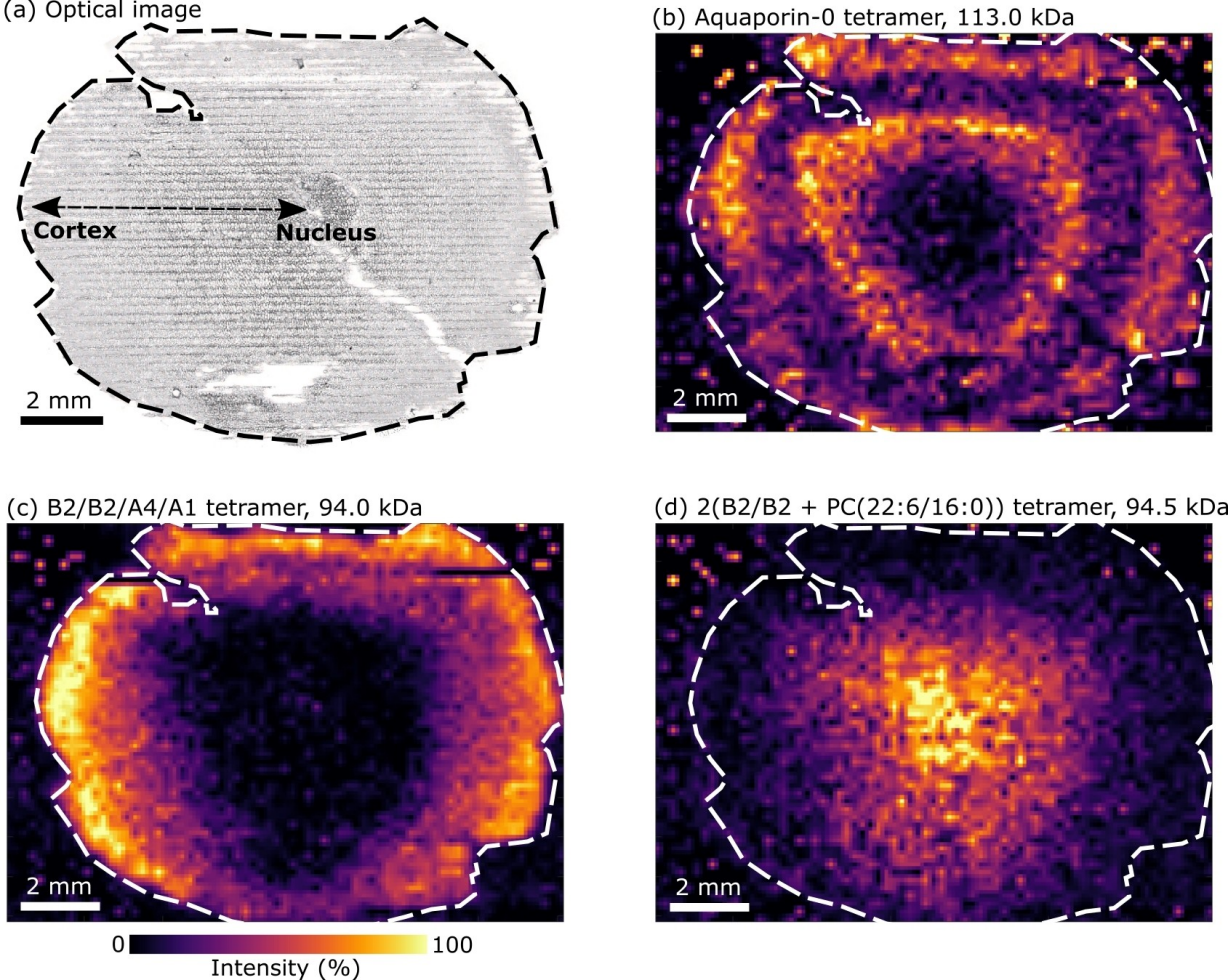
a) Optical image of the analysed lens tissue section. Nucleus tissue is central to the section and cortex tissue surrounds the nucleus. Lines are visible from the scanning of the nano‐DESI probe. b) Ion image of Aqp0 tetramer (113.1 kDa, *m*/*z* 6651.5^17+^ ±0.2). c) Ion image of B2/B2/A4/A1 crystallin tetramer (94.0 kDa, *m*/*z* 6267.3^15+^ ±0.2). d) Ion image of 2(B2/B2+PC (16:0/22:6)) crystallin tetramer (94.5 kDa, *m*/*z* 6299.2^15+^ ±0.2). Ion images are TIC normalised with a linear intensity scale and feature a pixel size of 200×200 μm.

Our results suggest that the conditions required for analysis of the tetrameric Aqp0 membrane protein remain suitable for analysis of soluble protein complexes. Crystallins are abundant structural lens proteins classified into three groups; α‐ β‐ and γ.^[33]^ Studies utilising MALDI detected α‐crystallins after sample treatment with denaturing solvents, but they were not detected here, potentially owing to low solubility in aqueous solvent. Signals for γ‐crystallin were detected, but only for the monomeric protein. The β‐crystallins are further distinguished as either basic “β‐B” or acidic “β‐A”. β‐B2‐crystallin assembles into oligomers with itself, and other basic and acidic β‐crystallins.[^34^, ^35^] Tetramers of β‐crystallins form by the association of β‐crystallin dimers.[^34^, ^36^] In addition to the identification and mapping of Aqp0, we identified tetrameric complexes of β‐crystallins (Figure 2c, d, Figures S8–S10, Supporting Information).

The heterotetrameric assembly B2/B2/A4/A1 is approx. 94.0 kDa in mass and comprises two β‐B2‐crystallin subunits, one β‐A4‐crystallin subunit and one β‐A1‐crystallin subunit (Figure 3a and b, Table S2, Figure S11, Supporting Information). In principle, the B2/B2/A4/A1 tetramer could be assembled via either B2/A4 and B2/A1 dimers or via B2/B2 and A4/A1 dimers. Dimers B2/B2, B2/A4 and B2/A1 were detected in the full scan mass spectra and their identities confirmed by collisional activation and dissociation to monomers (Figure S12, Supporting Information). Monomer identifications using sequence ions obtained by collisional activation of B2 monomers or B2/A4 and B2/A1 heterodimers are shown in Figures S13–S15 and Tables S3–S5, Supporting Information. The A4/A1 dimer was not detected and heterodimers of acidic β‐crystallins are known to only weakly associate,^[37]^ which suggests the tetramer assembles from B2/A4 and B2/A1 dimers. The B2/B2/A4/A1 tetramer was distributed with greatest abundance at the periphery of lens cortex tissue sections (Figure 2c), with a sharp decrease in intensity with increasing lens tissue age (i.e., towards the nucleus).

**Figure 3:**
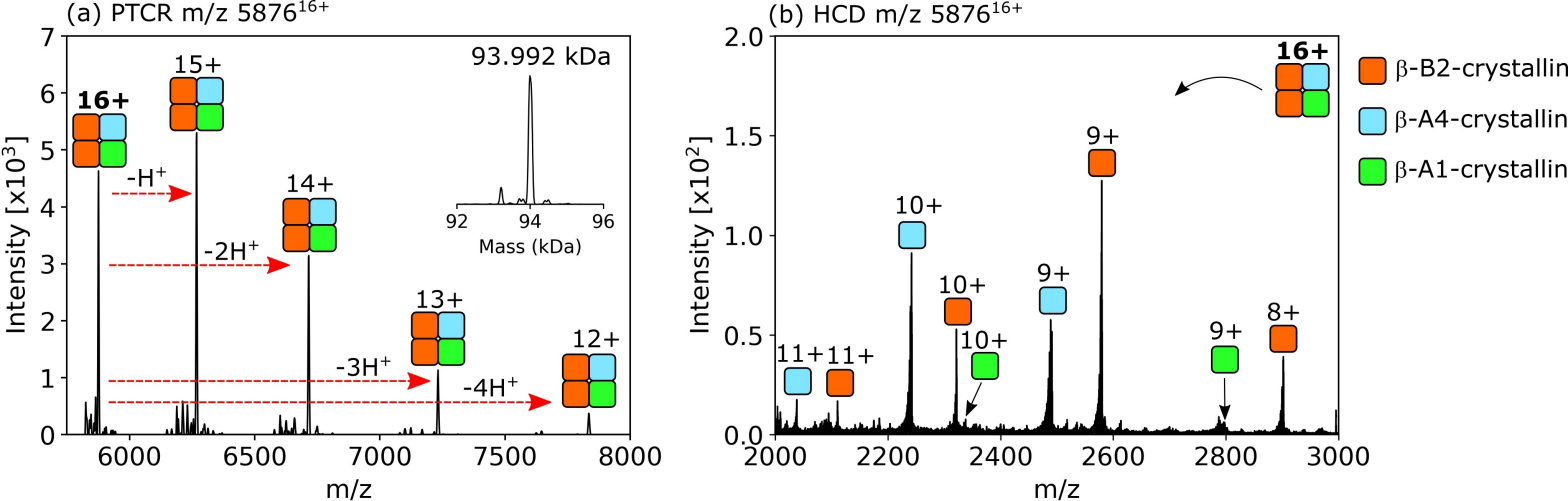
Identification of a 94 kDa crystallin heterotetrameric complex (a) Determination of the average mass of the complex by PTCR MS^2^ (93.992 kDa). b) HCD MS^2^ spectrum revealed three subunits; β‐B2, β‐A4 and β‐A1. The tetramer is composed from B2/B2/A4/A1 subunits. The sum of the measured average masses of the subunits is 94.001 kDa (calculated average mass 94.003 kDa).

Conversely, a 94.5 kDa assembly (Figure 4a) consisting of four N‐terminally acetylated βB2 subunits and two ligands showed the opposite spatial distribution, i.e., greatest abundance within the lens nucleus and absence in the lens cortex (Figure 2d). Gas‐phase dissociation of this assembly yielded signals for an approx. 47.2 kDa subunit, and βB2 monomers (Figure 4b). Interestingly, a peak corresponding to a mass loss of approx. 1600 Da from the intact tetramer was observed. Further collisional activation of the 47.2 kDa subunit resulted in product ions which suggest it comprises an intact βB2 homodimer plus an unidentified ligand of molecular weight approx. 805.5 Da (Figure 4c). We propose, therefore, that this tetramer is assembled from two βB2 dimers, each bound to a ligand of 805.5 Da. Higher energy collisional activation of the ligand‐bound dimer resulted in dissociation of the complex to βB2 monomers, as well as backbone fragmentation within both monomer and dimer (cleavage of the first three amino acid residues, i.e., acetyl‐ASD, MW 316.1 Da, leaving the y_201_ ion, residues 4‐204), and importantly, cleavage within the bound ligand (Figure 4d). The mass spectrum in Figure 4e focuses on bond cleavage within the bound ligand and may be interpreted in order to propose an identification, as in our previous work where the GDP ligand bound to the protein Arf3 fragmented by a separate pathway to the free GDP ions.^[8]^ A peak corresponding to the βB2 monomer plus intact ligand is not observed; however, a peak is observed with Δ*m*=328±1 Da, approximately the molecular weight of docosahexaenoic acid (C22 : 6). The remaining 478±1 Da of mass may be accounted for by a phospholipid headgroup and palmitic acid. Both of these fatty acids are known constituents of lens fibre cell membranes and changes in their abundance over time has been associated with cataract formation.[^38^, ^39^] We therefore suggest a tentative ligand identity of phosphatidylcholine PC(16:0/22 : 6) (805.56 Da), see Figure 4f. Direct detection and fragmentation of the ligand ions could confirm the ligand identity but efforts to this point have proven unsuccessful, likely due to a combination of low initial ion abundance, ion transmission inefficiency and ligand lability. (We note that positively charged phospholipid ions have been dissociated and analysed from protein‐lipid assemblies in purified solutions.^[7]^) Interaction of the β‐crystallins with the headgroups of membrane lipids has been suggested in a previous study.^[40]^ While a homotetrameric structure has been reported for β‐B2‐crystallin before (by x‐ray crystallography),^[34]^ this is the first report of endogenous tetrameric structure and ligand interaction.

**Figure 4:**
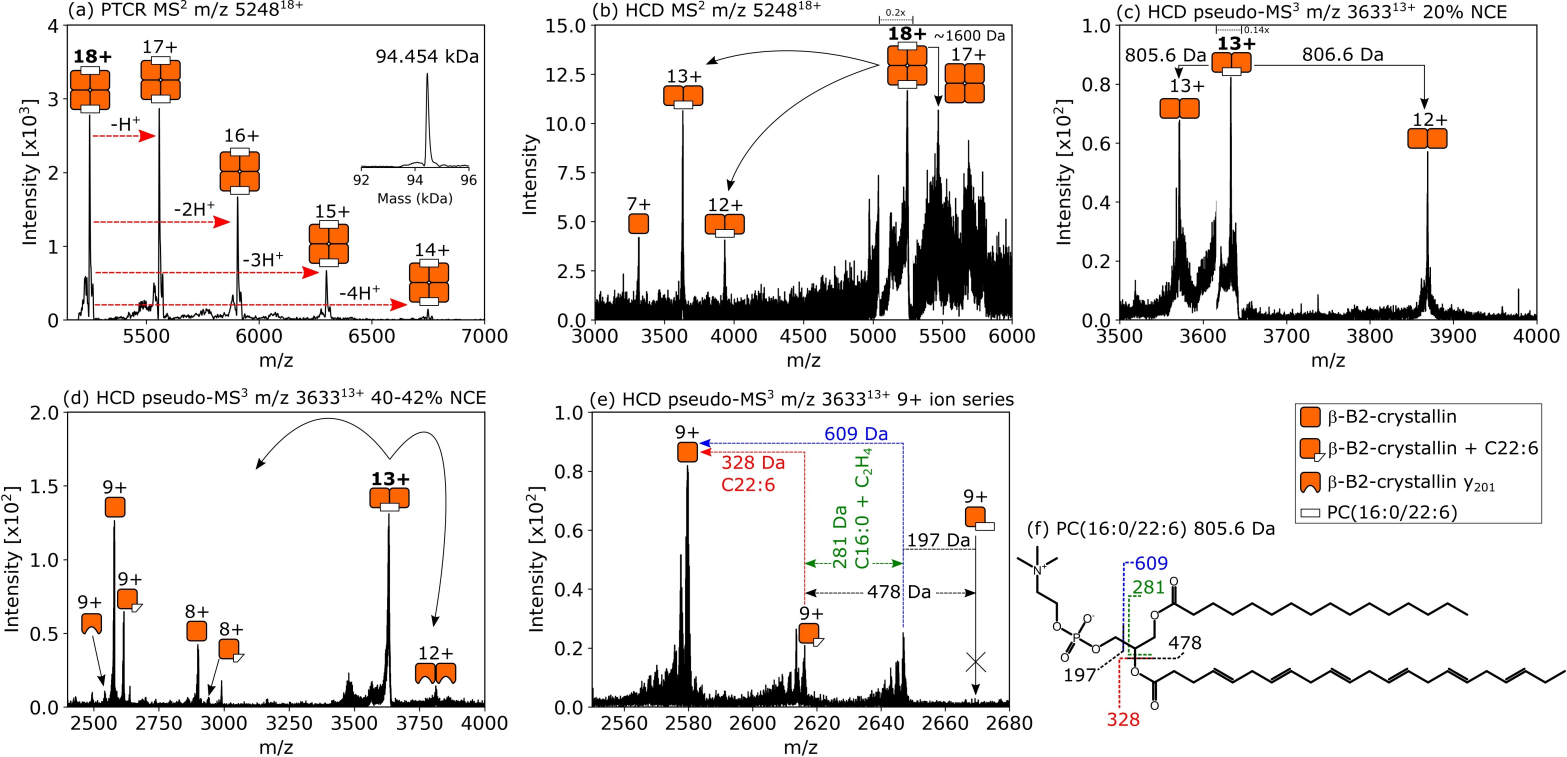
a) nano‐DESI PTCR MS^2^ spectrum showing charge reduction of the 18+ precursor ions (*m*/*z* 5248). The inset mass spectrum shows the deconvoluted mass as 94.454 kDa. b) HCD MS^2^ spectrum for *m*/*z* 5248^18+^. Signals were observed corresponding to 7+ ions of β‐B2‐crystallin, plus dimers of β‐B2‐crystallin with additional mass corresponding to approx. 806 Da in the 12+ and 13+ charge states, and a peak corresponding to loss of approx. 1600 Da from the intact tetramer. c) Evidence for neutral loss of ≈805.5 Da was obtained by pseudo‐MS^3^ of the dimer, *m*/*z* 3633^13+^; [*M*+H]^+^ (806.6 Da, 13+ → 12+) and neutral loss (805.5 Da, 13+ → 13+). These product ions are 2x the mass of β‐B2‐crystallin monomer detected in (b). d) Higher‐energy collisions resulted in additional fragmentation of the dimer, *m*/*z* 3633^13+^. The 12+ product ion signal indicates cleavage within the β‐B2‐crystallin subunits leaving a homodimer of y_201_ ions. A series of 9+ product ion signals include β‐B2‐crystallin, β‐B2‐crystallin 4‐204 (i.e. y_201_^9+^) and [β‐B2‐crystallin+327.7 Da]. e) Analysis focusing on the 9+ ion series. Mass differences were tentatively assigned to fragments of PC(16:0/22 : 6) as illustrated in (f). Peaks to the left of labelled peaks in (e) are indicative of neutral loss of water or ammonia, approx. −18 Da, during HCD.

The intact membrane protein assembly Aqp0 was identified and characterised directly from a thin tissue section by native ambient mass spectrometry. The sampling technique enabled spatial analysis of the intact assembly, highlighting its abundance in lens cortex tissue. We also characterised β‐crystallin complexes with molecular weight approx. 94 kDa and found evidence of the homotetrameric β‐B2‐crystallin assembly bound to phospholipid ligands.

Aqp0 is an abundant membrane protein and provided a functional test for the analytical method, which we expect to be broadly applicable to *in situ* membrane protein analysis in future work. Still, to advance this analysis approach we envisage developments on multiple fronts are required; 1) different membrane proteins “prefer” different detergents and so methods will likely need tailoring. Other available membrane mimetics such as amphipols and nanodiscs may also be considered, and new generation detergents may vastly improve membrane protein extraction;^[41]^ 2) non‐disruptive/denaturing tissue washing may enhance membrane protein signal intensity by reducing competition from abundant analytes during ionisation. The effects of tissue washing in native ambient MSI have yet to be explored; 3) high‐resolution mass isolation of precursor ions. Advances in this area of mass spectrometer functionality will be critical to resolve proteoforms from one another, and could improve sensitivity through targeted experiments, e.g., selected ion monitoring (SIM).

## Conflict of interest

The authors declare no conflict of interest.

## Supporting information

As a service to our authors and readers, this journal provides supporting information supplied by the authors. Such materials are peer reviewed and may be re‐organized for online delivery, but are not copy‐edited or typeset. Technical support issues arising from supporting information (other than missing files) should be addressed to the authors.

Supporting Information

## Data Availability

Data supporting this work is available from https://doi.org/10.25500/edata.bham.00000840.
